# Improving the resilience of maritime supply chains: The integration of ports and inland transporters in duopoly markets

**DOI:** 10.1007/s42524-022-0231-3

**Published:** 2022-12-22

**Authors:** Jia Shi, Jihong Chen, Lang Xu, Zhongjie Di, Qunzhen Qu

**Affiliations:** 1 College of Management, Shenzhen University, Shenzhen, 18071 China; 2 School of Transport and Communications, Shanghai Maritime University, Shanghai, 201306 China; 3 Hainan Special Project, Wuhan University of Technology, Sanya, 572025 China; 4 School of Economics & Management, Shanghai Maritime University, Shanghai, 201306 China

**Keywords:** resilience, Hotelling model, social welfare, maritime supply chain, game theory

## Abstract

The adverse impact of the outbreak of COVID-19 has reduced ports’ operational efficiency. In addition, ports and inland logistics providers are generally independent of each other and difficult to work together, which leads to time loss. Thus, as the core player, ports can integrate with inland logistics providers to improve the efficiency and resilience of maritime supply chains. This study examines the strategic options of two competing maritime supply chains consisting of ports and inland logistics providers. We investigate the impact of cooperation between ports and inland logistics providers and government regulation on the maritime supply chain by comparing members’ optimal pricing and overall social welfare under centralized, decentralized, and hybrid scenarios. Results indicate that the hybrid scenario is an equilibrium strategy for maritime supply chain, although this strategy is not optimal for governments seeking to improve supply chain resilience and maximize social welfare. Furthermore, observations show that through government economic intervention, both seaborne supplies can be incentivized to adopt an integrated strategy, and business and society can achieve a win–win situation.

This research is supported by the major projects of National Social Science Fund of China (Grant No. 20&ZD130).

## 1 Introduction

Since the outbreak of COVID-19 in January 2020, governments worldwide have adopted prevention and control policies to prevent the spread of the virus. The focus of the policies are on restricting gatherings and suspending work and production. However, the uncertainty brought about by the pandemic and the shortage of personnel and reduced efficiency due to the restrictions have put the global supply chain under enormous pressure and the risk of disruption. Ocean transportation is the main bearer of international trade, but it is one of the main ways for the virus to spread across borders. Thus, sea transportation and inland transportation are heavily restricted by the government's epidemic prevention measures (Xu et al., 2021). As a result, the collection and distribution efficiency of ports is greatly reduced, and a large number of goods are accumulated in port yards. In the post-pandemic era, economic recovery is the main task of countries. As the main bearer of international trade, 80% of the world's goods are completed by ocean transportation (Monios and Wilmsmeier, 2012; Chen et al., 2020a). Therefore, ensuring the stability of the maritime supply chain and improving its resilience is one of the conditions for promoting rapid economic recovery (Chen et al., 2019d). After the pandemic outbreak, the current structure of the maritime supply chain exposed problems. In particular, ports cannot communicate well and engage in collaborative operation with upstream and downstream enterprises. In the past, in order to ease the fierce competition and reduce logistics costs, most ports choose to outsource inland transportation services to logistics service providers, forming a decentralized maritime supply chain structure (Fig. 1). However, due to

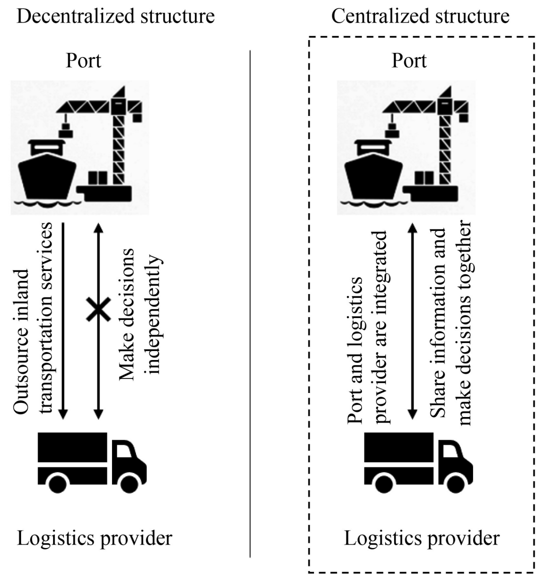

**Fig. 1** Decentralized/Centralized maritime supply chain structure.

the poor connection of each link in the transportation process and the low degree of information sharing, service efficiency is low and the resource waste is serious, thereby aggregating the double marginalization effect (Franc and van der Horst, 2010). During the pandemic, the maritime supply chain with a decentralized structure is more fragile, which is not conducive to ensuring the stability of the global supply chain.

The traditional hinterland transportation system includes many links, and many enterprises are involved in the logistics activities. A reasonable coordination plan lacks for hinterland transport, which affects the overall efficiency of the maritime supply chain and makes the hinterland collecting and distributing system a weak link in the maritime supply chain. To improve the resilience of the maritime supply chain, as the core of the maritime supply chain, ports can choose related companies that integrate the maritime supply chain (Scholten and Schilder, 2015; Liu et al., 2020), that is, forming a centralized supply chain structure (Fig. 1). A maritime supply chain with a centralized structure can strengthen the information communication between ports and inland logistics providers, and coordinate personnel and equipment to formulate more reasonable collection and distribution plans for goods. These advantages can ensure that the maritime supply chain can recover quickly in the event of a shock. At present, some port operators have begun to integrate inland logistics resources. For example, in August 2020, port operator DP World acquired once-based Avana Logistek, thereby consolidating their supply chain resources. UAE-based Abu Dhabi Ports has further expanded its logistics services by acquiring local logistics operator MICCO Logistics in September 2020. Furthermore, our research is based on a duopoly market, which is a common phenomenon in port competition. For example, Shanghai Port and Ningbo Port have overlapping hinterlands, as do Shenzhen Port and Hong Kong Port. In a duopoly market, where one maritime supply chain adopts a centralized structure and the other chooses a decentralized structure, we call this a hybrid scenario.

In addition to relying on enterprises' own strength to improve the resilience of the maritime supply chain, the experience of domestic and foreign countries has shown that the development of transport industries cannot be independent of government support (Witkowski and Kiba-Janiak, 2014; Chen et al., 2019a). As an industry with a high association effect, logistics enterprises have been hampered by individual goals. Hence, the government should strengthen their support in finance, taxation, and enterprise financing. Especially in times of economic reconstruction, the stability and efficiency of the maritime supply chain in the post-epidemic era is crucial for economic recovery and social development because it is the main bearer of international trade. For example, the US government claimed in 2022 that it plans to charge shippers a surcharge for containers stranded in port yards to ease the growing port congestion in the ports of Los Angeles and Long Beach. The purpose of the extra charges is to urge shippers to complete the delivery work as soon as possible and to speed up the flow of containers, and these charges will be used to improve port infrastructure and increase port capacity. Although previous studies found that the integration of ports and logistics service providers will increase the profits of ports, it may reduce social welfare (Álvarez-SanJaime et al., 2015), which ignores the different competitive scenarios in a duopoly market. Hence, our research is focused on the following research questions.

(1) In a market without government intervention, which strategy will the two maritime supply chains adopt? And is there an equilibrium strategy to achieve the equilibrium?

(2) What factors can promote the integration of ports and logistics providers, thereby improving their resilience? How do these factors influence enterprise profit and social welfare?

(3) Can corporate profits and maritime supply chain structure be optimized at the same time? If not, can the government intervene economically to achieve port integration with inland transporters?

To investigate these questions, we establish a Hotelling model with two maritime supply chains. According to this structure, to incorporate the impact of price competition, we assume that the relationship between ports and logistics providers can make decisions simultaneously to obtain a Nash–Stackelberg equilibrium. Beyond that, we explore the impact on the competition structure by assuming that the hinterland maritime supply chain has three structures: A centralized structure, a decentralized structure, and a hybrid structure. Besides, we consider the time cost to measure the possible additional losses in the decentralized structure. After deriving the equilibrium solutions of the above structures, we discuss the interactions between maritime supply chains when they choose the different structures and obtain the subgame perfect equilibrium.

From the derived observations, we obtain managerial insights into the pricing strategies of maritime supply chains. First, we construct the duopoly assumption where two ports are located at opposite points of the coastline to observe that both maritime supply chains choosing a centralized structure is not a Nash equilibrium of the game where two ports decide simultaneously whether to integrate in an initial stage. We then conclude that one maritime supply chain taking the centralized structure and the other taking the decentralized one is a stable marketing configuration without government regulation. In addition, we consider the strategic-level question for government whether the integration of the maritime supply chain can be promoted without reducing the profits of the maritime supply chain. With regard to the government regulation, we obtain an interesting observation that social welfare can be improved without reducing the profits of chains by government intervention. As extra taxes increase, the profits of chains under government regulation rise constantly.

The remainder of the paper is organized as follows. We review the related literature in Section 2. The problem description and model analysis are introduced in Section 3. We discuss the government strategy in Section 4. In Section 5, we conduct the numerical analysis. Finally, the conclusions are provided in Section 6.

## 2 Literature review

Our research lies at the interaction of supply chain resilience, port competition and inland transport, and pricing strategies. In this section, we describe how our work closely relates to the literature in the aforementioned areas.

### 2.1 Supply chain resilience

In recent years, terrorist incidents and major natural disasters have adversely affected supply chains (Wagner and Bode, 2008; Scholten et al., 2014). In particular, the current COVID-19 pandemic has increased uncertainty and the risk of disruption to global supply chains (Aslam et al., 2020). Research has then shifted to supply chain resilience. Supply chain resilience is defined as "the capability of a supply chain to develop the required level of readiness, response, and recovery capability to manage disruptions risks, and get back to the original state or even a better state after disruptions" (Chowdhury et al., 2019). The ability of a supply chain to withstand turbulence, disruption, and unforeseen events is important to all supply chain managers (Bhamra et al., 2011; Brandon-Jones et al., 2014). When the supply chains of enterprises have strong resilience, once risks are unavoidable, they can allocate internal resources and systems, and be fully prepared to overcome the threat of interruption and continue to provide goods and services to customers (Ponomarov and Holcomb, 2009; Ambulkar et al., 2015; Tukamuhabwa et al., 2017; Sahebjamnia et al., 2018).

Scholars believe that the key to forming resilience is the integration and coordination of human and material resources in the supply chain (Jüttner and Maklan, 2011; Scholten and Schilder, 2015). Many authors have found that collaboration, joint planning, and enhanced information exchange among supply chain members can help create synergies and recover from supply chain disruptions quickly (Cao et al., 2010; Wieland and Wallenburg, 2013). The literature on supply chain resilience suggests that environmental uncertainty and disruption are not confined to organizational boundaries. Instead, they affect the entire supply chain (Stoll et al., 2015; Ali et al., 2017). Therefore, for core enterprises, cooperation or integration with upstream and downstream members can strengthen decision synchronization and incentive alignment, which are essential for improving supply chain resilience (Daugherty et al., 2006; Ponomarov and Holcomb, 2009; Chowdhury and Quaddus, 2017).

### 2.2 Port competition and inland transport

Another related subsection is the literature on port competition. For port competition, we review the literature on non-spatial game theory and Hotelling model used in port competition (Kaselimi et al., 2011; Luo et al., 2012; Asgari et al., 2013; Homsombat et al., 2013; Tian et al., 2015; Dong et al., 2016; Zhang et al., 2017). Do et al. (2015) developed an uncertain payoff two-person game model where an uncertain factor of demand is involved. Song et al. (2016) established a non-cooperative game model of a two-port-one-sea carrier system and deduced the optimal ports' pricing and the carrier's port-of-call decisions.

The Hotelling model is one of the most famous spatial game models, and it is applicable to this topic. Hotelling (1929) argued that competition between services provided at different locations is oligopolistic in nature because of the importance of transportation costs. In the maritime supply chain, the cost of inland transportation is one of the main factors for shippers to choose ports. The differentiation of transported products satisfies the basic assumptions of the Hotelling model. Kaselimi and Ishii first applied the Hotelling model to the study of port competition. Kaselimi et al. (2011) introduced a horizontal product differentiation model to analyze the competition among container terminals. They analyzed a landlord port management system with long-term concession agreements that shape the formal relationship between the port authority and private terminal operators. Ishii et al. (2013) constructed a non-cooperative game model in which each port strategically selects port charges according to the timing of port capacity investment, and applied these methods to the competition between the ports of Busan and Kobe. A growing number of researchers are conducting studies on port competition by using Hotelling model (Homsombat et al., 2013). Zhou (2015) constructed a two-dimension Hotelling model to analyze the ports between competition and cooperation. Álvarez-SanJaime et al. (2015) established a Hotelling model between ports and investigated the economic incentives and welfare implications to the integration of ports with inland transport services. A number of studies on port competition based on emission control have emerged to determine whether to use the Hotelling model or the non-spatial game theory (Cui and Notteboom, 2017; Sheng et al., 2017; Liu et al., 2019a; Peng et al., 2019). On this basis, Randrianarisoa and Zhang (2019) used a two-stage game model to solve the problem of whether to invest in ports in the context of climate change. Further, Xu et al. (2018) considered carbon dioxide emissions and used the Logit model to describe the discrete selection behavior of shippers and to build a game theory model of port competition.

Our research is also related to inland transport. Literature on hinterland transportation generally proceeds in two perspectives: Empirical method (Monios and Wilmsmeier, 2013; Álvarez-SanJaime et al., 2015; Zhang et al., 2017; Chen et al., 2020b; Yin et al., 2021) and numerical analysis (Sugawara, 2017; Luo and Chang, 2019). The integration of port and inland transportation is the main trend of port supply chain, and many studies have been conducted on this subject (Wang and Liu, 2019). Tan et al. (2018) investigated the vertical integration between ocean carrier and inland shipping company. However, a large number of scholars have done research related to intermodal transport (Witte et al., 2017; Gonzalez Aregall et al., 2018; Zhang et al., 2018; Liu et al., 2019b) and hinterland transport (Frémont and Franc 2010; Chen et al., 2019b; 2019c; 2020c; Hua et al., 2020) from the perspective of environment protection. Li et al. (2015) studied the planning of intermodal transport between ocean terminal and inland terminal by container transport operators. Woodburn (2017) examined the trends in the operational efficiency of the container rail freight market in the UK port hinterland and assessed the impact of any changes on the overall sustainability.

Most of the above literature consider the impact of inland transportation on port supply chains, mainly focusing on the impact of port pricing and site selection. However, only a few scholars focus on the influence of hinterland maritime supply chain on port competition. This article examines the competition between maritime supply chains in three cases (i.e., decentralized game, hybrid game, and centralized game) for areas not covered previously.

### 2.3 Pricing strategies

Price plays a significant role in hinterland maritime supply chain under competition structure (Merkel, 2017; Xu et al., 2018; Peng et al., 2020; Xie et al., 2021). Compared with aforementioned literature, few researchers have focused on the pricing strategies under competition structure. de Borger et al. (2008) focused on the relationship between freight decision and investment strategy for port in the hinterland transport. Luo et al. (2012) considered a two-stage duopoly model to obtain the decisions of price and capacity allocation in an increasing sea-cargo market. King et al. (2014) adopted empirical analysis to discuss how the pricing strategies of hinterland transport impacts the forwarder with regard to regional port. Xu et al. (2015) aimed at the coordination and investigated the influence of market demand on forwarding freight of regional ports. Meersman et al. (2016) aimed at a micro-research method to investigate how the price decision influences the cost in the maritime supply chain and how it affects the competitiveness of regional ports. Yang et al. (2019) analyzed the effect of port integration in a region on the pricing strategies in the scenario when the port supply is higher than demand. Wang and Liu (2019) provided the revenue and cost sharing mechanism to investigate the pricing strategies and coordinate the maritime supply chain.

From the perspective of maritime supply chain, Song (2002) investigated and compared the pricing strategies between centralized and decentralized maritime supply chains. On this basis, Wang et al. (2017) and Xu (2020) combined the horizontal and vertical competition, respectively, to investigate the price decision with two-to-one chain through duopoly competition in sea-cargo demand. Further, Sheng et al. (2017) considered the competition structure between a shipping company and a port in a Stackelberg–Nash game to reason the price equilibria. Notwithstanding, the literature mostly focuses on pricing decision, yet ignores the participants' perspective. Unlike in existing studies (e.g., Liu et al. (2019a)), this study not only considers the freight fee and forwarding fee, but also introduces participants' behaviors into the decision of sea-cargo market.

In contrast to each of the above-mentioned research, our observations focus on which competition structure the maritime supply chains will prefer. Further, the literature showed that in the competitive environment, the environmental concerns of stakeholders will lead to a decline of market competitiveness and will always benefit the ports closer to the market. Therefore, on this basis, we analyze the competition structure of maritime supply chain by using the Hotelling model. Moreover, we discuss how the pricing strategies encourage the competing chains to answer the question: Is there a way to improve social welfare without reducing the profits of maritime supply chains?

At present, one type of literature mainly analyzes the effect of the integration of ports and inland transportation services and solves the problem of inland port competition. Another category of related literature focuses on how to improve supply chain resilience, which can be summarized in Table 1. The above-mentioned studies show that most scholars use the Hotelling model to investigate the competition between two ports under different competition structures. Thus, it is necessary to supplement and continue the previous studies on port competition and integration of port and inland transportation. Specifically, we construct an enhanced Hotelling model with two maritime supply chains and analyze the outcomes under three different cooperation–competition scenarios. Moreover, we examine the role of government in the competition of maritime supply chains.

## 3 Problem description and model analysis

In this paper, we study the pricing and integration strategies of maritime supply chains in a duopoly market. Suppose that in a duopoly market, two ports are in the identical hinterland and compete for gateway freight in the overlapping market. In addition, two logistics providers transport goods from shippers to ports, where each logistics provider serves only one port. The port and logistics provider will choose whether to cooperate or not with the goal of maximizing total profits. Under this structure, we consider two logistics providers take actions simultaneously as followers, and the ports are the Stackelberg game leaders with equal bargaining power. Therefore, we adopt the backward induction to solve the Nash–Stackelberg game.

We construct the duopoly assumption where two ports (A and B) are located at opposite points of the coastline, as described in Fig. 2. Shippers are uniformly distributed in the interval [0, 1], which have an option on either maritime supply chain A1 or B2. For simplification, both ports provide a homogeneous service at an equal and constant cost, and corresponding logistics service providers serve as a bridge between shippers and port to transport. Given that the hinterland service is homogenous, the shipper chooses the port that quotes the least delivered price, namely, the service charge, distance expense, and congestion cost.

Without loss of generality, the congestion cost at port $i$ ($i = \mathrm{A, B}$) mainly depends on the portion of demand $x_i$ and service capacity $K_i$ with a linear function of $d'x_i/K_i$, where $d'$ is unit congestion cost. Referring to Sheng et al. (2017), we consider the scenario in which both ports provide the same capacity, that is, $K_\mathrm{A} = K_\mathrm{B} = K$, and the congestion cost is transferred as $dx_i$, where $d = d'/K_i$. Let $p_i$ and $s_j$ ($j = 1, 2$) be the charge of each port and logistics service provider, respectively. Hence, a shipper located at $x$ receives payoff $U_\mathrm{A} = v - p_\mathrm{A} - s_1 x - dx_\mathrm{A}$ if the shipper chooses from port A and $U_\mathrm{B} = v - p_\mathrm{B} - s_2(1-x) - dx_\mathrm{B}$ when the shipper chooses from port B, where $v$ indicates the utility of choosing the homogeneous service. $v$ is large enough that every owner will consider, whether

**Table 1** Gap between existing literature and our research

| | Supply chain resilience | Inland competition | Port competition | Chain competition |
|---|---|---|---|---|
| de Borger et al., 2008; Li and Oh, 2010; Asgari et al., 2013; Liu et al., 2019a | | √ | √ | |
| Ambulkar et al., 2015; Ali et al., 2017; Aslam et al., 2020 | √ | | | |
| Jüttner and Maklan, 2011; King et al., 2014; Dong et al., 2016 | | | √ | |
| Li and Oh, 2010; Li et al., 2015; Sheng et al., 2017; Hua et al., 2020 | | | √ | √ |
| Our research | √ | √ | √ | √ |

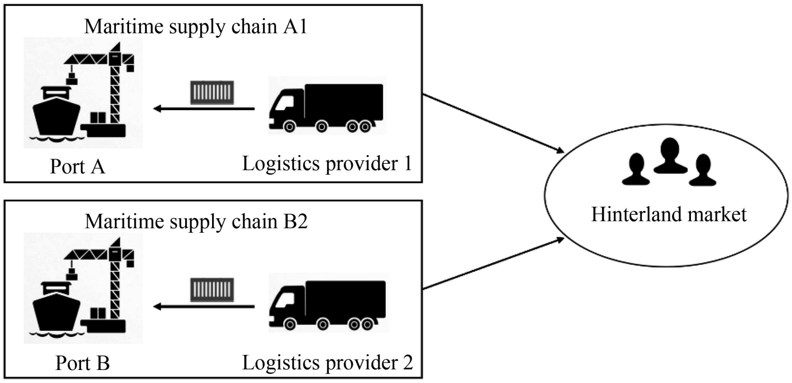

**Fig. 2** Structure between maritime supply chains.

from port A or B. The indifferent point $\hat{x}$ chosen from either port is decided by $U_A(\hat{x}) = U_B(\hat{x})$. With each owner having unity demand, port A's demand is $D_A = \hat{x} = x_A$ and port B's demand is $D_B = 1 - \hat{x} = x_B$. Simple calculations yield that $D_i = (d - p_i + p_j + s_j)/(2d + s_i + s_j)$.

### 3.1 Decentralized competition game

We first consider the case in which both maritime supply chains are decentralized, that is, ports and logistics service providers determine their charges respectively to maximize their profits. To examine the time loss of provider caused by checking and handling procedures between the port and the logistics provider, we introduce parameter $\delta$, which represents the time cost per unit of goods borne by logistics provider. We use superscript "$z$" to indicate that both chains are decentralized. Each port $i$ and logistics provider $j$ maximizes their (operating) profit $\pi_i^z = (p_i^z - h)D_i^z$, $\pi_j^z = s_j^z\int_0^{x_i} x\mathrm{d}x - \delta D_i^z = s_j^z(x_i^z)^2/2 - \delta D_i^z$ by choosing $p_i^z$ and $s_j^z$, respectively, where $h$ stands for the costs of the port service per freight unit. If we assume that ports and logistics providers simultaneously choose prices to maximize their profits, and the solution to $\partial\pi_i^z/\partial p_i^z = 0$, $\partial\pi_j^z/\partial s_j^z = 0$, for $i = $ A, B and $j = 1, 2$, then the following equilibrium can be yielded:

$$p_A^{z*} = p_B^{z*} = h + \frac{\sqrt{d(d+2\delta)} - d}{3}, \tag{1}$$

$$s_1^{z*} = s_2^{z*} = \sqrt{d(d+2\delta)} - \frac{4d}{3}, \tag{2}$$

where stars are used to denote the corresponding equilibrium. According to the equilibrium prices, we derive the demand of ports is $D_A^z = D_B^z = 1/2$, because the capacity and cost for two ports are assumed to be the same. Hence, the price difference between two maritime supply chains is zero, and each chain takes up half of the market. Profits under decentralized competition game are as follows:

$$\pi_A^{z*} = \pi_B^{z*} = \frac{\sqrt{d(d+2\delta)} - d}{3}, \tag{3}$$

$$\pi_1^{z*} = \pi_2^{z*} = \frac{\sqrt{d(d+2\delta)}}{8} - \frac{d}{12} - \frac{\delta}{2}. \tag{4}$$

To study the impact of maritime supply chain on the whole society and provide suggestions for government regulation, we construct social welfare expressions, $SW^z$, by combining profits of ports and logistics providers and shipper surplus:

$$SW^z = \pi_A^z + \pi_B^z + \pi_1^z + \pi_2^z + \int_0^{x_A^z}(v - p_A - s_1 x - d\hat{x})\mathrm{d}x + \int_0^{x_B^z}(v - p_B - s_2(1-x) - d(1-\hat{x}))\mathrm{d}x. \tag{5}$$

**Proposition 1.** When both two maritime supply chains are decentralized, the equilibrium port charges increase if the time cost and congestion cost rise (i.e., $\partial p^{z*}/\partial\delta > 0$, $\partial p^{z*}/\partial d > 0$), that is, a higher level of disutility leads to higher port charges. The freight charges increase with the increase of time cost, but their response to congestion cost is not constant.

**Proof:**

$$\frac{\partial p^{z*}}{\partial\delta} = \frac{d}{3\sqrt{d(d+2\delta)}} > 0, \tag{6}$$

$$\frac{\partial p^{z*}}{\partial d} = \frac{1}{3}\left(\frac{d+\delta}{\sqrt{d(d+2\delta)}} - 1\right) > \frac{1}{3}\left(\frac{d+\delta}{\sqrt{(d+\delta)(d+\delta)}} - 1\right) = 0, \tag{7}$$

$$\frac{\partial s^{z*}}{\partial\delta} = \frac{d}{\sqrt{d(d+2\delta)}} > 0, \tag{8}$$

$$\frac{\partial s^{z*}}{\partial d} = \frac{d+\delta}{\sqrt{d(d+2\delta)}} - \frac{4}{3}, \tag{9}$$

when $d \in (0, (4\sqrt{7}\delta - 7\delta)/7)$, $(d+\delta)/\sqrt{d(d+2\delta)} - 4/3 > 0$.

Figure 3 presents numerical examples to illustrate how charges of ports change with respect to time cost and congestion cost. For the purposes of illustration, we let the value of time cost $\delta$ change from 0 to 0.3. The horizontal axis is the value of time cost, and the vertical axis is port charge. The four lines represent different congestion costs for logistics service provider $j$, with $d = 0.10$, 0.12, 0.14, 0.16, respectively. The main observations are:

- For a given congestion cost $d$, the port charge increases as time cost $\delta$ rises. For example, if $d = 0.14$, when the time cost $\delta$ increases from 0 to 0.3, the port charge rises by 40 percent.
- For a given time cost $\delta$, the port charge increases with the rise of congestion cost $d$. For every 0.02 increase in congestion cost, the port charge will rise by about 0.008.

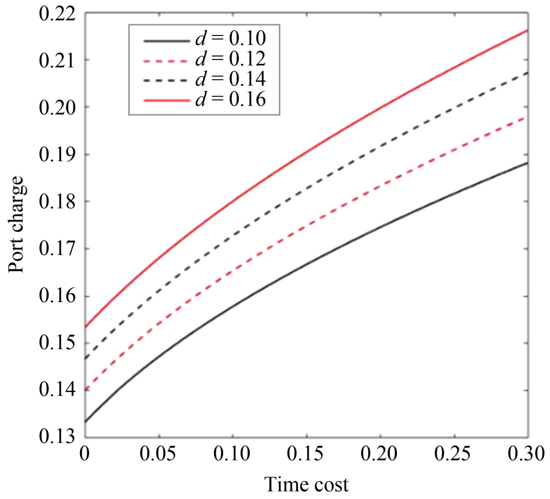

**Fig. 3** Port charge in decentralized competition game.

**Proposition 2.** When both two maritime supply chains are decentralized, the equilibrium profits of ports increase with the rise in port time cost and congestion cost; for logistics providers, the profits decrease as time cost rises and increase as the congestion cost rises (i.e., $\partial\pi^{z*}_{\mathrm{A/B}}/\partial\delta>0$; $\partial\pi^{z*}_{\mathrm{A/B}}/\partial d>0$; $\partial\pi^{z*}_{1/2}/\partial\delta<0$; $\partial\pi^{z*}_{1/2}/\partial d>0$). Thus, disutility does not necessarily harm the profits of ports and logistics providers.

**Proof:**

$$\frac{\partial\pi^{z*}_{\mathrm{A/B}}}{\partial\delta}=\frac{d}{3\sqrt{d(d+2\delta)}}>0, \tag{10}$$

$$\frac{\partial\pi^{z*}_{\mathrm{A/B}}}{\partial d}=\frac{1}{3}\left(\frac{d+\delta}{\sqrt{d(d+2\delta)}}-1\right)>\frac{1}{3}\left(\frac{d+\delta}{\sqrt{(d+\delta)(d+\delta)}}-1\right)=0, \tag{11}$$

$$\frac{\partial\pi^{z*}_{1/2}}{\partial\delta}=\frac{1}{8}\left(\frac{d}{\sqrt{d(d+2\delta)}}-4\right)<\frac{1}{8}\left(\frac{d}{\sqrt{d(d+0)}}-4\right)<0, \tag{12}$$

$$\frac{\partial\pi^{z*}_{1/2}}{\partial d}=\frac{1}{24}\left(\frac{3(d+\delta)}{\sqrt{d(d+2\delta)}}-2\right)>\frac{1}{24}\left(\frac{3(d+\delta)}{\sqrt{2}(d+\delta)}-2\right)>0. \tag{13}$$

Figures 4 and 5 present numerical examples to illustrate how profits of ports or logistics providers change with respect to time cost and congestion cost. The horizontal axis in both figures is the value of time cost. In Fig. 4, the vertical axis is the port's profit, whereas in Fig. 5, the vertical axis is the profit of the logistics service provider. The four lines represent different congestion costs for logistics service provider $j$, with $d=0.10$, 0.12, 0.14, 0.16, respectively. The main observations are:

● For a given congestion cost $d$, the profit of port increases slowly as time cost $\delta$ rises. For example, if $d=0.14$, when the time cost $\delta$ increases from 0 to 0.3, the profit of port rises from 0.0235 to 0.0533. The profit of logistics service provider decreases drastically as time cost $\delta$ increases. For example, if $d=0.14$, when the time cost $\delta$ increases from 0 to 0.01, the profit of logistics provider drops from 0.0058 to 0.0021.

● The profits of ports and logistics service providers increase with the rise of congestion cost $d$. The main reason for this increase is their pricing increases as $d$ rises, but the market share does not decrease.

### 3.2 Hybrid competition game

In this section, we study the case where one maritime supply chain is centralized while the other is not. We show how to decide optimal charges of ports and logistics service providers in the decentralized chain and centralized chain. We also study how the equilibrium prices and profits change compared with the decentralized competition game. Further, we determine whether the centralized

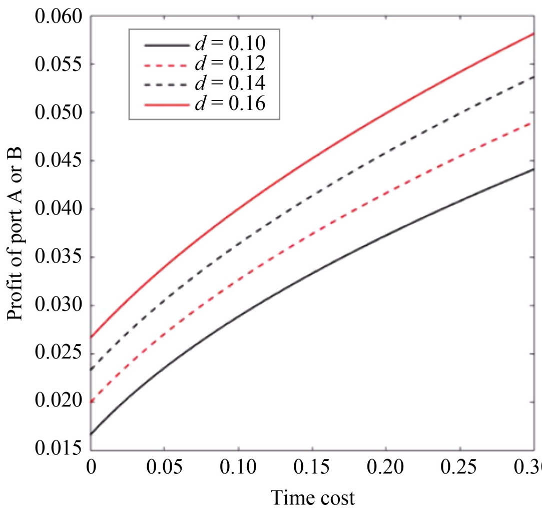

**Fig. 4** Profit of port A or B in decentralized competition game.

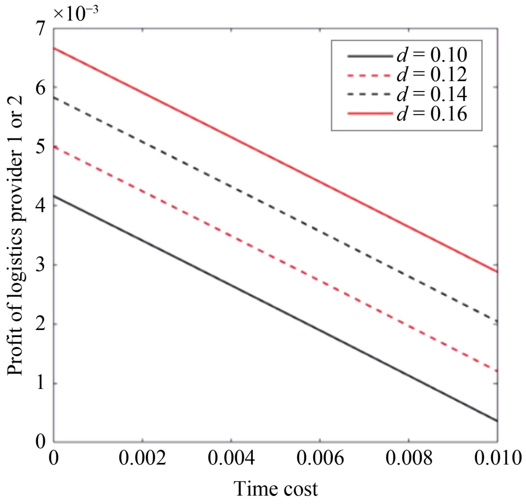

**Fig. 5** Profit of logistics provider 1 or 2 in decentralized competition game.

maritime supply chain has stronger competitiveness than decentralized chain does in this hybrid competition game. Without loss of generality, we assume the maritime supply chain with port A and logistics provider 1 is centralized. We use the superscript "$y$" to denote the hybrid competition game. Given that port A and logistics provider 1 have been allied, logistics provider 1 does not have to bear the time cost. The total profit of centralized maritime supply chain is $\pi^{y}_{\mathrm{A},1}=(p^{y}_{\mathrm{A}}-h)D^{y}_{\mathrm{A}}+(x^{y}_{\mathrm{A}})^{2}s^{y}_{1}/2$. Port B's profit is $\pi^{y}_{\mathrm{B}}=(p^{y}_{\mathrm{B}}-h)D^{y}_{\mathrm{B}}$, and the profit of logistics provider 2 is $\pi^{y}_{2}=s^{y}_{2}(x^{y}_{\mathrm{B}})^{2}/2-\delta D^{y}_{\mathrm{B}}$. The goal of port A and logistics provider 1 is to maximize the total profit of the centralized maritime supply chain, whereas port B and logistics provider 2 want to maximize their individual profits.

Let us focus on the first order condition for centralized maritime supply chain. In order to discover the relationship between $s^{y}_{1}$ and $\pi^{y}_{\mathrm{A},1}$, we calculate $\partial\pi^{y}_{\mathrm{A},1}/\partial s^{y}_{1}$:

$$\frac{\partial \pi_{A,1}^{y}}{\partial s_1^{y}} = \frac{(d+s_1^{y}-3p_A^{y}+p_B^{y}+2h)(d+s_2^{y}-p_A^{y}+p_B^{y})}{2(2d+s_1^{y}+s_2^{y})} - \frac{s_1^{y}(d+s_2^{y}-p_A^{y}+p_B^{y})}{(2d+s_1^{y}+s_2^{y})^2}. \quad (14)$$

It is negative under sufficient condition that $(s_2^{y}+p_B^{y}+2h+1)/3 < s_1^{y} < s_2^{y}+p_B^{y}+1$. If we assume that two maritime supply chains simultaneously choose prices to maximize profits given $s_1^{y}=0$, the solution to $\partial\pi_{A,1}^{y}/\partial p_A^{y}=0$, $\partial\pi_B^{y}/\partial p_B^{y}=0$ and $\partial\pi_2^{y}/\partial s_2^{y}=0$, the following equilibrium can be yielded:

$$p_A^{y*} = \frac{2d+2\delta+\sqrt{(6\delta+5d)^2-24\delta d}}{3}+h, \quad (15)$$

$$p_B^{y*} = \frac{5d+\sqrt{(6\delta+5d)^2-24\delta d}}{6}+h+\delta, \quad (16)$$

$$s_1^{y*}=0, \quad (17)$$

$$s_2^{y*} = 3\delta+\frac{\sqrt{(6\delta+5d)^2-24\delta d}-d}{2}. \quad (18)$$

Hence, the demands at equilibrium are given by $D_A^{y} = (12\delta+2\sqrt{(6\delta+5d)^2-24\delta d}+4d)/(18\delta+3\sqrt{(6\delta+5d)^2-24\delta d}+9d)$, $D_B^{y}=1-D_A^{y}$. Compared with the initial situation, the demand of centralized maritime supply chain has increased, because $D_A^{y}+D_B^{y}=1$, $D_A^{y}>D_B^{y}$, $D_A^{y}>1/2$. The effect of $s_1^{y}$ being smaller than $s^{z}$ is to steal shipper demand from port B leading at equilibrium to a flat line, $s_1^{y*}=0$. Shippers who are far choose port A for the lowest cost, so the centralized chains enlarge their market share. Finally, equilibrium profits read:

$$\pi_{A,1}^{y*} = \frac{2\left(2d+6\delta+\sqrt{(6\delta+5d)^2-24\delta d}\right)^2}{9\left(3d+6\delta+\sqrt{(6\delta+5d)^2-24\delta d}\right)}, \quad (19)$$

$$\pi_B^{y*} = \frac{\left(5d+6\delta+\sqrt{(6\delta+5d)^2-24\delta d}\right)^2}{18\left(3d+6\delta+\sqrt{(6\delta+5d)^2-24\delta d}\right)}, \quad (20)$$

$$\pi_2^{y*} = \frac{\left(6\delta-d+\sqrt{(6\delta+5d)^2-24\delta d}\right)\left(5d+6\delta+\sqrt{(6\delta+5d)^2-24\delta d}\right)}{6\left(3d+6\delta+\sqrt{(6\delta+5d)^2-24\delta d}\right)}. \quad (21)$$

**Proposition 3.** When port A integrates with logistics service provider 1, the equilibrium price and profit of centralized maritime supply chain increase with the rise in time cost of decentralized maritime supply chain (i.e., $\partial p_A^{y*}/\partial\delta > 0$, $\partial\pi_{A,1}^{y*}/\partial\delta > 0$).

**Proof:**

$$\frac{\partial p_A^{y*}}{\partial\delta} = \frac{2}{3}+\frac{6(d+2\delta)}{\sqrt{25d^2+36d\delta+36\delta^2}} > 0, \quad (22)$$

$$\frac{\partial \pi_{A,1}^{y*}}{\partial\delta} = \frac{17d+34\delta+5\sqrt{25d^2+36d\delta+36\delta^2}}{4\sqrt{25d^2+36d\delta+36\delta^2}} > 0. \quad (23)$$

Next, we compare the equilibrium prices and the total profit of each maritime supply chain under the hybrid competition game with those in the decentralized competition game. We obtain the following result.

**Proposition 4.** In the hybrid competition game, the equilibrium prices of port A, B and logistics provider 2 are greater than those in the decentralized competition game, that is, $p_A^{y*} > p_A^{z*}$, $p_B^{y*} > p_B^{z*}$, $s_2^{y*} > s_2^{z*}$; whereas the charge of logistics provider 1 is smaller (i.e., $s_1^{y*} < s_1^{z*}$).

**Proof:**

$$p_A^{y*}-p_A^{z*} = d+\frac{2\delta}{3}-\frac{\sqrt{d(d+2\delta)}}{3}+\frac{\sqrt{25d^2+36d\delta+36\delta^2}}{3} > 0, \quad (24)$$

$$p_B^{y*}-p_B^{z*} = \frac{7d}{6}+\delta-\frac{\sqrt{d(d+2\delta)}}{3}+\frac{\sqrt{25d^2+36d\delta+36\delta^2}}{6} > 0, \quad (25)$$

$$s_2^{y*}-s_2^{z*} = \frac{5d}{6}+3\delta-\sqrt{d(d+2\delta)}+\frac{\sqrt{25d^2+36d\delta+36\delta^2}}{2} > 0. \quad (26)$$

Figure 6 presents a numerical example of **Proposition 4**. For illustration purposes, we let $d=0.05$ and $h=0.1$. The vertical axis in the figure represents the equilibrium price of ports, and the horizontal axis is the value of time cost. The three lines represent different structures of maritime supply chains.

**Proposition 5.** In the hybrid competition game, the total profits of whole maritime supply chains are greater than those in the decentralized competition game, that is, $\pi_{A,1}^{y*} > \pi_A^{z*}+\pi_1^{z*}$, $\pi_B^{y*}+\pi_2^{y*} > \pi_A^{z*}+\pi_1^{z*}$.

Figure 7 presents a numerical example of **Proposition 5**, where the $x$-axis is the value of time cost $\delta$ and the $y$-axis is the maritime supply chain's profit. We assume $d=0.1$. The black solid curve and the red dashed curve represent the total profits of the marine supply chain A1 and B2 in the hybrid competition game, respectively; the red solid curve represents the total profit of either chain in the decentralized competition game (given that the two chains are completely symmetric, either chain's profits are the same as the other chain). Figure 8 shows the equilibrium in the decentralized competition game and the equilibrium when one maritime supply chain is centralized. The height at each point on the line indicates the full price incurred by the shipper located there. The black solid lines represent the decentralized competition game where the full price intersects at the midpoint. The red solid lines represent hybrid competition game where the

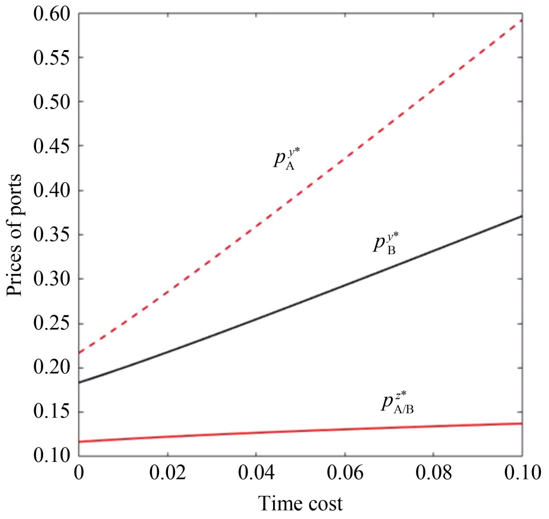

**Fig. 6** Comparison of equilibrium prices.

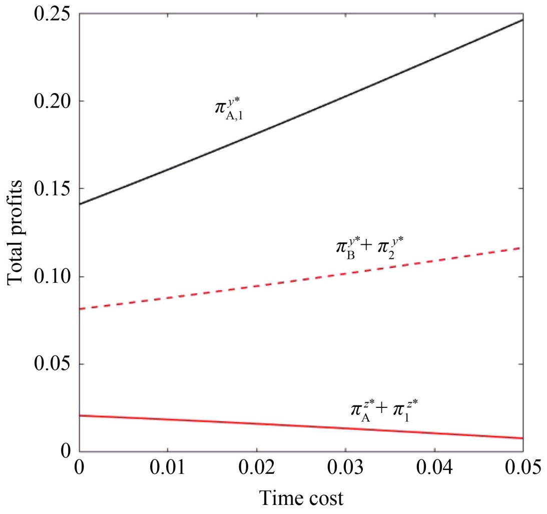

**Fig. 7** Comparison of total profits of whole maritime supply chains.

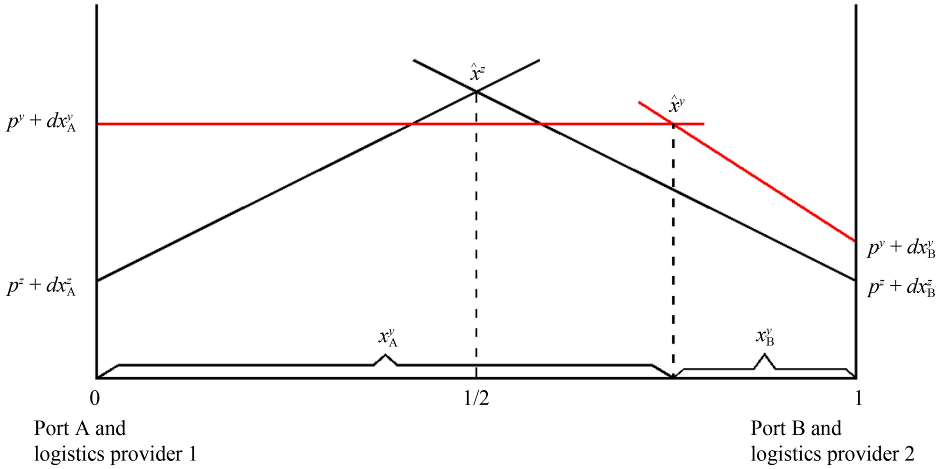

**Fig. 8** Comparison of port demand equilibrium allocation.

full price does not increase with the distance from port A. **Proposition 6** shows the main results.

**Proposition 6.** The port has unilateral incentive to integrate with the logistics service provider, that is, $\pi_{A,1}^{y*} > \pi_A^{z*} + \pi_1^{z*}$. Then the centralized maritime supply chain has a competitive advantage over the decentralized chain in this hybrid competition game, that is, $\pi_{A,1}^{y*} > \pi_B^{y*} + \pi_2^{y*}$.

**Proof:** The difference between the total profit of port A and logistics provider 1 in hybrid competitive game and decentralized competitive game is given by:

$$\pi_{A,1}^{y*} - \pi_A^{z*} - \pi_1^{z*} = \frac{5d}{12} + \frac{\delta}{2} - \frac{11\sqrt{d(d+2\delta)}}{24} + \frac{2\left(2d+6\delta+\sqrt{(6\delta+5d)^2-24\delta d}\right)^2}{9\left(3d+6\delta+\sqrt{(6\delta+5d)^2-24\delta d}\right)}. \tag{27}$$

The profit difference between centralized maritime supply chain and decentralized maritime supply chain in the hybrid competitive game is given by:

$$\pi_{A,1}^{y*} - \pi_B^{y*} - \pi_2^{y*} = \frac{88d\beta + 228\delta\beta + 1212d\delta + 536d^2 + 1368\delta^2 - (21\beta + 63d + 126\delta)\sqrt{d(d+2\delta)}}{72(3d+6\delta+\beta)},$$

$$\beta = \sqrt{(6\delta+5d)^2 - 24\delta d}. \tag{28}$$

According to the basic inequality, $\sqrt{d(d+2\delta)} < d+\delta$, we can derive the following results:

$$\pi_{A,1}^{y*}-\pi_{A}^{z*}-\pi_{1}^{z*}>\frac{\left(\frac{2}{9}(2d+6\delta+\beta)-\frac{3d}{8}\right)(2d+6\delta+\beta)}{3d+6\delta+\beta}>0, \tag{29}$$

$$\pi_{A,1}^{y*}-\pi_{B}^{y*}-\pi_{2}^{y*}>\frac{473d^2+1023d\delta+67d\beta+1242\delta^2}{72(3d+6\delta+\beta)}>0. \tag{30}$$

Therefore, the centralized structure entitles port A and logistics service provider 1 to competitiveness advantage to expand their market shares as well as increase profit. The resulting increase in the revenue suffices to offset the increase in the transportation costs because demand increases. However, when $\delta\in(d(10-7\sqrt{2})/4, d(10+7\sqrt{2})/4)$, the social welfare decreases in the hybrid competitive game, that is, $SW^y<SW^z$. Thus, the reduction in shipper surplus is greater than the increase in corporate profits. On the one hand, shippers who are far from port A are better off because they do not have to pay for the inland transportation, which accounts for most of the total cost. On the other hand, shippers who are close to port A are worse off. Although they do not pay for the inland transportation, they have to pay the higher port fee and suffer the higher disutility due to higher congestion in port A. Hence, the change in shipper surplus is highly dependent on the shippers' location, leading to groups of shippers that are better off (those farther from the ports), whereas others are worse off (those closer to the ports).

### 3.3 Centralized competition game

Now, we study the case in which both maritime supply chains are centralized. In this section, the port and the logistics service provider are fully integrated to achieve the whole maritime supply chain's maximum revenue. We seek to derive the equilibrium price and profit of each maritime supply chain and compare the differences between the centralized competition game and the previous two settings. We use superscript "$t$" to denote the centralized competition game. The total profits of two maritime supply chains can be expressed by $\pi_{i,j}^t=(p_i^t-h)D_i^t+(D_i^t)^2s_j^t/2$. Considering that the profit function of the two maritime supply chains is the same, the prices and profits of the two maritime supply chains must be the same in equilibrium. For notation convenience, we denote $p_A^t=p_B^t=p^t$, $s_1^t=s_2^t=s^t$, and $\pi_{A,1}^t=\pi_{B,2}^t=\pi^t$. We find $\partial\pi^t/\partial s^t$ is always negative if $p^t>h+(s^t+1)/2$. Given that $s^{t*}=0$, assuming two maritime supply chains simultaneously decide port charges to maximize the profit of whole chains, and the solution to $\partial\pi_{A,1}^t/\partial p_A^t=0$, $\partial\pi_{B,2}^t/\partial p_B^t=0$, the following equilibrium can be obtained:

$$s_1^{t*}=s_2^{t*}=0, \tag{31}$$

$$p_A^{t*}=p_B^{t*}=d+h. \tag{32}$$

Demand at equilibrium in the centralized competition game is same as the demand in the decentralized competition game, that is, $D_A^{t*}=D_B^{t*}=1/2$. Bringing the above results into profit function, we obtain the profits and social welfare at equilibrium $\pi_{A,1}^{t*}=\pi_{B,2}^{t*}=d/2$, $SW^{t*}=v-d/2-h$.

**Proposition 7.** When one of the maritime supply chains has been integrated, the ports in the other maritime supply chain will not choose to integrate with logistics service providers for the purpose of maximizing profits. However, in order to achieve higher social welfare, the government has an incentive to turn both chains into a centralized structure.

**Proof:** The difference between the total profit of port B and logistics provider 2 in the hybrid competitive game and the centralized competitive game is given by:

$$\pi_B^{y*}+\pi_2^{y*}-\pi_{B,2}^{t*}=\frac{3\beta^2+17d^2\beta+180\delta^2\beta+2160d\delta^2+1584d^2\delta+388d^3+1728\delta^3+108d\delta\beta}{72\left(3d\beta+6\delta\beta+36d\delta+17d^2+36\delta^2\right)}>0. \tag{33}$$

Given that $\pi_B^{y*}+\pi_2^{y*}>\pi_{B,2}^{t*}$, when the maritime supply chains decide whether to integrate or not, both maritime supply chains choose centralized structure, which is not a Nash equilibrium of the game. We then conclude that a hybrid structure integrating the maritime supply chain and another decentralized structure is a stable market configuration without government regulation. Then we compare the social welfare under the three scenarios and find that $SW^t>SW^z>SW^y$. The social welfare difference between the centralized competition game and the decentralized competition game is $\delta$. As the general regulator, the government always expects the global profit to be maximized. Therefore, the government has an incentive to stimulate the two chains to adopt a centralized structure. According **Proposition 7**, the final equilibrium state is not the best result for the government. Is there a strategy that can take into account corporate profits and social welfare? We will discuss this question in next section.

## 4 Government regulation

In the previous section, we derived the optimal prices and profits for the decentralized, hybrid and centralized competition games. In this section, we consider the strategic-level question for the government: Is there a way to improve social welfare without reducing the profits of maritime supply chains?

Previous studies suggest that the government can subsidize ports by charging extra taxes when two maritime supply chains are both centralized (Zhang et al., 2017). To ensure fairness and increase the shipper surplus of shippers who are near ports, we suggest that the government should charge an extra tax $\alpha$ that is proportional to the distance to subsidize ports. Then a shipper located at $x$ receives payoff $U_A = v - p_A - (s_1 + \alpha)x - dx_A$ when the shipper chooses from port A and $U_B = v - p_B - (s_2 + \alpha)(1 - x) - d(1 - x_A)$ when the shipper chooses from port B. Similarly, from $v - p_A - (s_1 + \alpha)x - dx_A = v - p_B - (s_2 + \alpha)(1 - x) - d(1 - x_A)$, we obtain $D_i = (p_j - p_i + s_j + d)/(s_i + s_j + 2\alpha + 2d)$. The profits of two centralized maritime supply chains are given by:

$$\begin{aligned}\pi^k_{i,j} &= \left(p^k_i - h\right)D^k_i + s^k_j \int_0^{x^k_A} x\mathrm{d}x + \alpha \int_0^{x^k_A} x\mathrm{d}x \\ &= \left(p^k_i - h\right)D^k_i + \frac{s^k_j + \alpha}{2}\left(x^k_A\right)^2. \end{aligned} \tag{34}$$

The superscript "$k$" is used to denote the competition game with government regulation. Same to the centralized competition game, we let $p^k_A = p^k_B = p^k$, $s^k_1 = s^k_2 = s^k$, and $\pi^k_{A,1} = \pi^k_{B,2} = \pi^k$. We find that $\partial\pi^k/\partial s^k$ is always negative if $p^k > h + d + \alpha/2$. Given that $s^{k*} = 0$, assuming two maritime supply chains simultaneously decide port charges to maximize the profits of whole chains, and solve the equations $\partial\pi^k_{A,1}/\partial p^k_A = 0$, $\partial\pi^k_{B,2}/\partial p^k_B = 0$, the following equilibrium can be yielded:

$$s^{k*}_1 = s^{k*}_2 = 0, \tag{35}$$

$$p^k_A = p^k_B = h + d + \frac{\alpha}{2}. \tag{36}$$

When the government implements regulatory measures, the equilibrium profits of two chains turn to $\pi^{k*}_{A,1} = \pi^{k*}_{B,2} = d/2 + 3\alpha/8$. Social welfare at equilibrium is $SW^k = v + \alpha/4 - d/2 - h$.

**Proposition 8.** By charging appropriate additional fees, the government can improve social welfare without reducing the profits of maritime supply chains.

**Proof:** When the total profits of chains are the same as before, we can derive two thresholds of $\alpha$, which can be denoted as $\alpha^{k1}$ and $\alpha^{k2}$:

$$\pi^{k*}_{A,1} = \pi^{y*}_{A,1} \to \alpha^{k1} = \frac{16(2d + 6\delta + \beta)^2}{27(3d + 6\delta + \beta)} - \frac{4d}{3}, \tag{37}$$

$$\pi^{k*}_{B,2} = \pi^{y*}_B + \pi^{y*}_2 \to \alpha^{k2} = \frac{\beta(3\beta^2 + 17d^2 + 180\delta^2 + 108d\delta) + d^2(1584\delta + 388d) + \delta^2(2160d - 1728\delta)}{81d\beta + 162\delta\beta + 972d\delta}. \tag{38}$$

Let $\alpha^k = \max(\alpha^{k1}, \alpha^{k2})$, the profits of both maritime supply chains increase under government regulation if $\alpha > \alpha^k$. Then both maritime supply chains choosing centralized structure is a Nash equilibrium where ports decide simultaneously whether to integrate the hinterland maritime supply chain. The difference of social welfare in the centralized competition game and this section is $\alpha$, which is always positive. As per the above calculation results, social welfare can be improved without reducing the profits of chains by government intervention. Figure 9 provides a numerical example to illustrate **Proposition 8**. For illustration purposes, we let $d = h = 0.1$ and $\delta = 0.005$. The vertical axis represents the total profits of maritime supply chains, and the horizontal axis is the value of extra taxes. As the extra taxes increase, the profits of chains under government regulation rise constantly. Finally, the profits of chains under government regulation surpass those in the hybrid competition game.

We further investigate the thresholds of extra taxes with the change of the value of $d$. As shown in Figs. 10 and 11, both thresholds are positive with the value of $d$, that is, $\partial\alpha^{k1}/\partial d > 0$ and $\partial\alpha^{k2}/\partial d > 0$. Parameter $d$ represents the congestion degree of the port to some extent, which means that the government needs to set higher extra taxes to subsidize the port if the increase of cargo volume makes the port crowded. Moreover, the additional subsidies provided by the government need to be greater than the larger of the two thresholds. As $d$ increases, $\alpha^{k2}$ gradually exceeds $\alpha^{k1}$, becoming the bottom line for the extra tax. Besides, by assuming different time costs, we find that the region where $\alpha^{k1}$ is greater than $\alpha^{k2}$ extends under a larger time cost, but the value of the thresholds decreases overall.

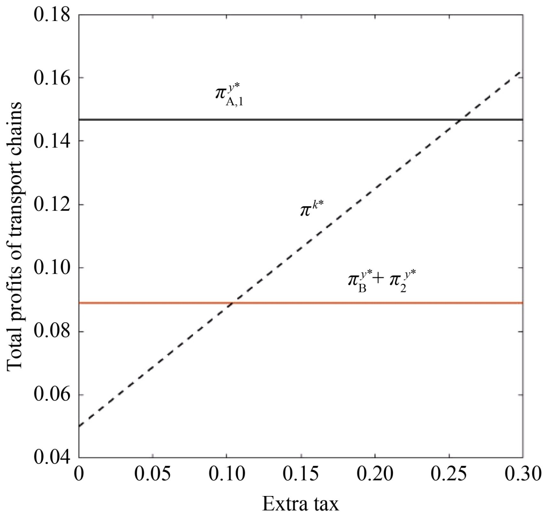

**Fig. 9** Comparison of total profits with or without government regulation.

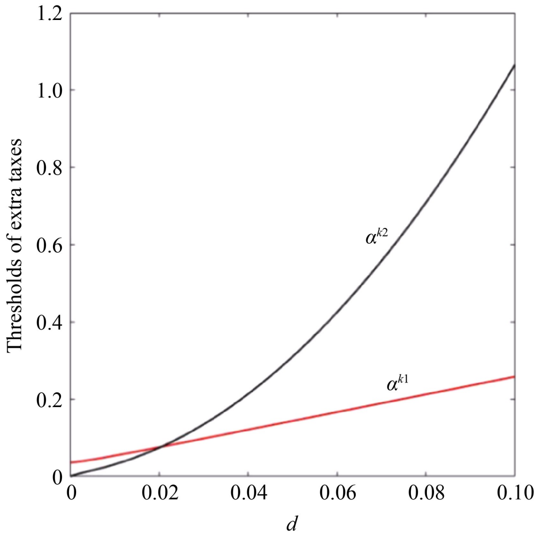

**Fig. 10** Threshold of extra taxes under $\delta = 0.005$.

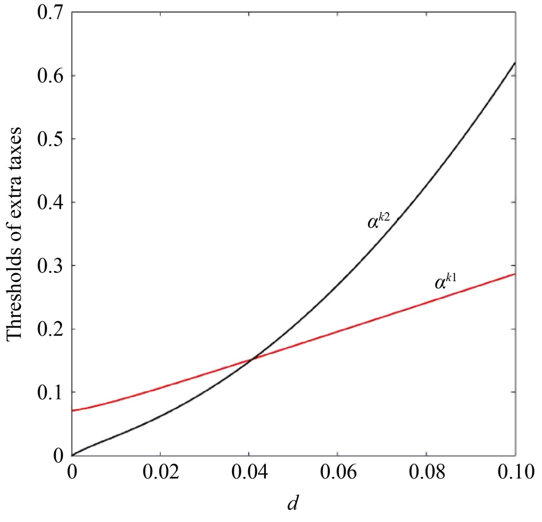

**Fig. 11** Threshold of extra taxes under $\delta = 0.01$.

## 5 Numerical analysis

In this section, we conduct numerical analysis on the basis of the previous theoretical analysis. For convenience, we define decentralized competition game, hybrid competition game, and centralized competition game as Scenario 1, 2, and 3, respectively. Considering the coefficient values used in the existing literature, the parameters of this paper are set as follows: $d = 0.1$, $h = 1$, $\delta = 0.005$. First, we calculate the optimal prices, profits, and social welfare under different scenarios, and provide their variation values among different structures of maritime supply chains. The results are presented in Table 2.

The first column of Table 2 shows the equilibrium variables obtained under three scenarios. The second, third, and fourth columns display the equilibrium results under Scenarios 1, 2, and 3, respectively. The main results in Table 2 are summarized as follows. By adopting a centralized maritime supply chain, the alliance between port A and logistics service provider 1 achieved the purpose of reducing cost and expanding market share, and the total profit of the maritime supply chain greatly increased compared with the previous scenario. Specifically, the demand of port A increased from 0.5000 to 0.5881, and the total profit of the maritime supply chain increased by 0.1269. Although the maritime supply chain A1 is more competitive than maritime supply chain B2, the profits of port B and logistics provider 2 were higher than before. This is because Port B increases port charges for shippers who are closer to it. If Port B also chooses to integrate with the logistics provider, then it will share the entire market equally with Port A. However, compared with Scenario 2, this strategy leads to a decrease in total maritime supply chain profit (−0.0967 for chain A1 and −0.0390 for chain B2) due to the reduction of port charges. Given that the increase in shipper surplus (0.1393) was greater than the decrease in corporate profits (−0.1357), the total social welfare rose by 0.0036.

**Table 2** Equilibrium results under three scenarios

| | Scenario 1 | Scenario 2 | Scenario 3 |
|---|---|---|---|
| Port **A**'s price | 0.1350 | 0.3495 | 0.2000 |
| Port **A**'s demand | 0.5000 | 0.5881 | 0.5000 |
| Port **A**'s profit | 0.0175 | | |
| Logistics provider **1**'s price | 0.0382 | | |
| Logistics provider **1**'s profit | 0.0023 | | |
| Chain **A1**'s profit | 0.0198 | 0.1467 | 0.0500 |
| Chain **A1**'s profits variation | | 0.1269 | −0.0967 |
| Port **B**'s price | 0.1350 | 0.2748 | 0.2000 |
| Port **B**'s demand | 0.5000 | 0.4119 | 0.5000 |
| Port **B**'s profit | 0.0175 | 0.0720 | |
| Logistics provider **2**'s price | 0.0382 | 0.2243 | |
| Logistics provider **2**'s profit | 0.0023 | 0.0170 | |
| Chain **B2**'s profit | 0.0198 | 0.0890 | 0.0500 |
| Chain **B2**'s profits variation | | 0.0692 | −0.0390 |
| Shipper surplus | $v - 0.1945$ | $v - 0.3893$ | $v - 0.2500$ |
| Shipper surplus variation | | −0.1948 | 0.1393 |
| Social welfare | $v - 0.1550$ | $v - 0.1536$ | $v - 0.1500$ |
| Social welfare variation | | 0.0014 | 0.0036 |

Next, we define competition game under government regulation as Scenario 4. Then the two thresholds of extra taxes are 0.2580 and 0.1976. For simplicity, we let $\alpha = 0.26$, and the results are presented in Table 3. The fourth column of Table 3 presents the difference of the equilibrium solutions between Scenarios 2 and 4.

The results in Table 3 show that under government intervention, maritime supply chain A1's total profit rose by 0.0008 and maritime supply chain B2's total profit

**Table 3** Equilibrium results under government regulation

| | Scenario 2 | Scenario 4 | Variation |
|---|---|---|---|
| Port **A**'s price | 0.3495 | 0.3300 | −0.0195 |
| Port **A**'s demand | 0.5881 | 0.5000 | −0.0881 |
| Port **A**'s profit | | | |
| Logistics provider **1**'s price | | | |
| Logistics provider **1**'s profit | | | |
| Chain **A1**'s profit | 0.1467 | 0.1475 | 0.0008 |
| Port **B**'s price | 0.2748 | 0.3300 | 0.0552 |
| Port **B**'s demand | 0.4119 | 0.5000 | 0.0881 |
| Port **B**'s profit | 0.0720 | | |
| Logistics provider **2**'s price | 0.2243 | | |
| Logistics provider **2**'s profit | 0.0170 | | |
| Chain **B2**'s profit | 0.0890 | 0.1475 | 0.0585 |
| Shipper surplus | $v - 0.3893$ | $v - 0.3800$ | 0.0093 |
| Social welfare | $v - 0.1536$ | $v - 0.0850$ | 0.0686 |

increased more (0.0585) by charging extra fees. The increase in profits indicates that two maritime supply chains have enough motivation to make a centralization decision in order to maximize profits. Owing to the government's regulation, the integration of the two maritime supply chains has been completed and the cost of time loss due to poor information exchange has been eliminated, which has reduced port A's charges by 0.0195. Therefore, the profits of the enterprise rose, and shipper surplus did not decrease, which has actually increased by 0.0093, so the total social welfare rose by 0.0686. The above results show that achieving a win–win situation for both enterprises and shippers cannot be done without the government's push. Under government supervision, two maritime supply chains shift toward a centralized structure and improve the resilience and service quality of maritime supply chain.

We make implications referring to the test results by combining the development models of some Asian ports. An extensive case survey of ports confirmed key features behind the rise of Asian hub ports over the last three decades. Unlike port developments in the UK and most of those in Europe, the development of Asian ports has been driven by the central government's multiple roles, including port designers, developers, operators, pricing makers, and investors; landside connectivity is also part of the integrated planning under the central government (Tae-Woo Lee and Flynn, 2011). The early stages of port development require large investments, such as port and industrial parks and electric utilities, which cannot be afforded by individual private companies. In addition, the functions of Asian container ports and large industrial ports handling liquefied natural gas and crude oil are closely related to the national economic development in the government's strategic economic development plan. During the pandemic period, in order to ensure the stability of the maritime supply chain, the government has taken measures by using economic or administrative means. For example, as the world's largest trading nation, China regards port services as public goods. To improve the resilience of the maritime supply chain and resist the impact of the pandemic, the Chinese government has given priority to the port logistics industry, and has formulated many preferential policies such as financial subsidies. Many ports in China, such as Shanghai Port and Ningbo–Zhoushan Port, have taken advantage of government investment to expand port capacity, integrate transport resources, and build a comprehensive multimodal maritime supply chain. Besides, in the first half of 2022, the US government planned to impose a congestion surcharge on shippers for containers stranded in port yards in an effort to alleviate growing port congestion. The purpose is to urge cargo owners to complete the pickup work as soon as possible and speed up the flow of containers. The fees collected will be used to improve port infrastructure and increase port capacity.

## 6 Conclusions and policy suggestions

### 6.1 Conclusions

Ensuring the stability and efficiency of the maritime supply chain in the post-epidemic era is crucial for economic recovery and social development because it is the main bearer of international trade. To improve the resilience of the maritime supply chain, as a core enterprise, ports can strengthen cooperation by integrating with inland transporters. On the one hand, the ports design and operate the maritime supply chain with the goal of maximizing their own profits. On the other hand, the government should maintain a balance between enterprises and society. In this paper, we conduct a systematic study of how to design and operate maritime supply chains to increase resilience and improve profitability. The pricing strategies for decentralized, centralized, and hybrid model are discussed, and the effect of government regulation is analyzed. Moreover, the following management insights are obtained. First, if both maritime supply chains are concentrated and provide the exact same services, then port congestion due to emergencies such as the pandemic will not significantly reduce profits. For logistics providers, the impact of time loss on profits is huge. Compared with ports, the logistics providers are more willing to seek alliances with ports.

Second, in the absence of government intervention, a much superior option is to maintain one of the decentralized maritime supply chains and the prices rise accordingly. However, to improve the resilience of the maritime supply chain, moving from a decentralized structure to a centralized structure as soon as possible is necessary, and social welfare may decline when the time loss is huge. Compared with the decentralized scenario, the centralized maritime supply chain can not only deal with the risk of interruption, but also has a competitive advantage, which can occupy more market shares and increase profits.

Lastly, governments could subsidize ports by charging shippers additional fees, thereby encouraging the integration of the two maritime supply chains. Under the supervision of the government, the centralized structure can not only improve corporate profits and social welfare, but also enhance the resilience and service quality of maritime supply chains.

### 6.2 Policy suggestions

In terms of improving the security and stability of the maritime supply chain, this paper puts forward three suggestions. First, the government needs to cultivate multimodal transport market players actively with the ability to integrate multiple logistics resources. The core enterprises in the supply chain must be guided to extend their service scope and transform into multimodal transport operators and integrated logistics service providers. Under the guidance of government departments, a cross-industry alliance composed of ports, shipping companies, and inland logistics providers can be established to improve the operational efficiency of the maritime supply chain through information collaboration and strategic interaction. The second is to ensure the overall stability of shipping logistics through the centralized structure of the supply chain. Especially in terms of supply chain integration, enterprises can establish broader business alliances according to the characteristics of demand distribution. A complementary cargo source collection network can be formed to alleviate port congestion caused by external risks. The third is to establish a risk response mechanism that can respond quickly. It is recommended to collect and organize all aspects of logistics data in the maritime supply chain to achieve cross-departmental sharing of information. Big data technology can be used to evaluate market fluctuations caused by uncontrollable factors such as the pandemic. In addition, the early warning mechanism can be used to suppress the spread of risks and control external costs.

Some issues need further study. For example, this paper only studies the competition and cooperation between identical maritime supply chains, whereas the different port capacities and transport costs remain to be explored. Additionally, the horizontal cooperation between maritime supply chains with uncertain markets needs to be studied.

**Open Access** This article is licensed under a Creative Commons Attribution 4.0 International License, which permits use, sharing, adaptation, distribution and reproduction in any medium or format, as long as you give appropriate credit to the original author(s) and the source, provide a link to the Creative Commons licence, and indicate if changes were made.

The images or other third party material in this article are included in the article's Creative Commons licence, unless indicated otherwise in a credit line to the material. If material is not included in the article's Creative Commons licence and your intended use is not permitted by statutory regulation or exceeds the permitted use, you will need to obtain permission directly from the copyright holder. To view a copy of this licence, visit http://creativecommons.org/licenses/by/4.0/.
